# The Use of Kinesthetic Empathy with Adults Living with Treatment Resistant Depression: A Survey Study

**DOI:** 10.1007/s10465-022-09371-4

**Published:** 2022-09-23

**Authors:** Neha Christopher, Jeanette Tamplin

**Affiliations:** 1 University of Melbourne, Melbourne, Australia; 2 Indian Association of Dance Movement Therapy, Chennai, Tamil Nadu India

**Keywords:** Kinesthetic empathy, Treatment-resistant depression, Dance/movement therapy, Regulation, Interoception

## Abstract

Dance/movement therapy is an embodied healing practice which has been found to foster recovery from depression and boost quality of life. Although kinesthetic empathy holds great potential for addressing emotional dysregulation, it is an under-utilized dance/movement therapy intervention in health optimization, especially in adults living with treatment-resistant depression. The aim of this study was to collect data from dance/movement therapist on how they use kinesthetic empathy to foster self-regulation in adults living with treatment-resistant depression. A survey design was used to obtain this data. Eight dance/movement therapists (practicing in India, Philippines, Barbados, and the United States of America) were recruited. The participant demographics of the survey challenges the centrality of master’s level trained dance/movement therapists primarily practicing in Eurocentric cultures. The survey included open answer questions, and responses received were grouped into four categories: (i) dance/movement therapists’ rationale for using kinesthetic empathy (ii) therapist-described client responses to kinesthetic empathy, (iii) changes in self-regulation patterns of clients, and (iv) potential links between employing kinesthetic empathy as an intervention and witnessing emotional regulation in clients. Overall, kinesthetic empathy was described as a core part of the participants’ dance/movement therapy practice with this population. The identified client responses to kinesthetic empathy were categorized based on deWitte’s et al. (Frontiers in Psychology, 2021) therapeutic factors of change. ‘Observable client responses’, such as, use of metaphors, verbalization of body sensations and engaging in mirroring were categorized under specific therapeutic factors of dance/movement therapy. Alternatively, ‘emotionally felt client responses’ such as, increase in safety and trust within the therapeutic alliance were categorized under both ‘specific’ and ‘mixed-type’ factors based on the model. Finally, this article discusses movement interventions that may be incorporated by dance/movement therapists while working with this population. Further research is required to identify the long-term effect/s of kinesthetic empathy as an intentional intervention to foster self-regulation in adults living with treatment-resistant depression.

**Supplementary Information:**

The online version contains supplementary material available at 10.1007/s10465-022-09371-4.

## Introduction

As an embodied healing practice, dance/movement therapy has been shown to alleviate symptoms of clinical depression (Karkou et al., 2019; Olmedo, 2020; Pylvänäinen et al., 2015). Within the last decade, there has been a steady increase in the global emergence of dance/movement therapy (Capello, 2016). Joining this momentum of robust and upcoming discoveries in dance/movement therapy, this article highlights a study aimed to explore dance/movement therapists’ *understanding*, and *use* of kinesthetic empathy while working with adults living with treatment-resistant depression in their practice.

While extensive research to discover the most effective treatment methods for depression has been ongoing for decades, a large component of this research remains to be on major depressive disorders. In comparison, little is known about the prognosis of treatment resistant depression (TRD). Currently, TRD still lacks a standardized and consensual definition (Rybak et al., 2021; Souery et al., 2006). Regardless of the scientific headway in mental health, depressive disorders continue to heavily burden the global public healthcare system, especially since the onset of the COVID-19 pandemic (Santomauro et al., 2021). Distinctive research about the use of a non-invasive and relational intervention like kinesthetic empathy towards symptom reduction and health optimization in adults living with TRD is crucial and necessary, especially with the expected rise in levels of global depression, post-traumatic stress, anxiety, and dysregulation following the COVID-19 pandemic (Ćosić et al., 2020).

The standardized use of dance/movement therapy in healthcare is in its nascent stages. Within depression treatment, prior research suggests that dance/movement therapy interventions can increase vitality and overall quality of life in adults (Karkou et al., 2019; Koch et al., 2007). Along with this, dance/movement therapy has also been found to address certain biochemical deficits in depression by modulating neurohormones, like serotonin and dopamine in teens with mild depression (Jeong et al., 2005). Current literature also suggests that clients have also benefited from dance/movement therapy’s positive effect on social symptoms of depression as it has been found to nurture a secure attachment style and increase emotional awareness in adults (Punkanen et al., 2014).

One of the lived experiences of depression is a difficulty in regulation (Joormann & Gotlib, 2010). Within TRD, dysregulation may be of emotional, social or of somatic nature. Trends in research and funding of healthcare suggest a disproportional distribution of resources between emotional and social dysregulation and somatic dysregulation. However, the risk that accompanies somatic dysregulation is no lower than that of emotional or social dysregulation. Consistent exposure to triggers causing subsequent dysregulation can even result in somatoform disorders like chronic fatigue or dissociative disorders (Bailer et al., 2017). While using somatic aptitude to address these forms of dysregulation may seem judicious from a theoretical framework in dance/movement therapy; it is not yet evidence based or well-researched. Somatic aptitude, like TRD, lacks a universally accepted definition. In practice, the authors understand somatic aptitude to include recognizing, navigating, and coping with sensory experiences and cues. Common somatic cues include, tingling, stomach churning, muscle tightness, chronic pain, etc., typically in response to emotional triggers (Friedrichsdorf et al., 2016; Trivedi, 2004).

Although kinesthetic empathy is a central aspect of dance/movement therapy, it is not unique to the field. Kinesthetic empathy is also experienced in social interaction, in performance studies, and in several creative practices across the world (Reynolds & Reason, 2012). A motif across these diverse areas is the affirmation that kinesthetic empathy is both a relational and somatic experience. A key aspect in understanding kinesthetic empathy is acknowledging its roots in emotioncy, which in-turn refers to emotional awareness though sensory intelligence (Pishghadam et al., 2013). This both justifies the union of kinesthetic empathy and sensory intelligence and highlights a new avenue to potentially nurture emotional awareness and self-regulation. The link between them is under-researched in dance/movement therapy. In this study, we present how kinesthetic empathy is being used as an intervention by dance/movement therapists, specifically to foster self-regulation in individuals living with TRD.

### The Complexity of Treatment Resistant Depression (TRD)

Based on the medical model, treatment resistant depression (TRD) is best understood as a state where depressive symptoms do not decrease despite at least two completed rounds of antidepressants (Al-Harbi, 2012a). It involves periods of low mood with physiological and psychological symptoms (Pandarakalam, 2018, pp. 280–281). Due to the complex nature of depression, reasons for treatment failure differ on an individual case-by-case basis. McIntyre et al. (2020) acknowledge the lack of time psychiatrists can offer versus the time needed to identify a well-rounded health plan as a plausible cause for treatment failure. This, along with an overall lack of illness acceptance, poor doctor-patient relationship, and medical non-compliance are other factors noted for failure in treatment (Hirschfeld, 2001; McIntyre et al., 2020). The lack of a strength-based and client-centred treatment model is evident in these research studies. On the contrary, although dance/movement therapy is a strength-based approach (Okamoto, 2017) more research is required for the anticipated benefits of this approach to be researched to address the multi-layered needs of adults living with treatment resistant depression.

### Kinesthetic Empathy and Regulation

Depending on one’s theoretical framework, kinesthetic empathy maybe either perceived as intentional intervention or everyday social phenomena. In this study, kinesthetic empathy has been positioned to be an intervention actively used by dance/movement therapists to foster self-regulation. Theories of intersubjectivity support kinesthetic empathy as a naturalistic process occurring in the milieu by claiming  “the co-presence of bodies in intersubjective situations can give rise to processes of kinesthetic empathy”. Alternatively, kinesthetic empathy may also be perceived as an intentional movement imitation process that can be facilitated in certain settings such as in dance classes (Koehne et al., 2016).

Despite differences in ways of understanding kinesthetic empathy, certain similarities include kinesthetic empathy being relational in nature and including embodiment. The former might include relational or interpersonal exchanges between two or more individuals or, between performers and their audience or, between therapists and their clients. Similarly, embodiment can also be a relational experience and be perceived differently based on one’s chosen worldview. However, a difference between the two is that unlike kinesthetic empathy, embodiment can occur in the absence of another individual whereas kinesthetic empathy cannot. Beaudoin and Maclennan (2021) describe embodiment as tangible knowledge that is gathered through the body-brain connection. Embodiment plays a vital role in dance/movement therapy as it is through embodiment that a therapist might gain insight into their clients’ emotional state and via embodiment that clients might also begin to identify and comprehend the emotional cues coupled with their sensory experiences. This section deliberately highlights differing viewpoints on, embodiment and kinesthetic empathy. While they might add richness of information in some disciplines, this lack of consensual information also serves as an added challenge in the use of non-invasive somatic practices like embodiment and kinesthetic empathy in treatment planning for adults living with treatment resistant depression (TRD). The authors of this article have also chosen not to refer any one central definition of kinesthetic empathy and in doing so pointing readers towards the clear impetus for future dance/movement therapy research in this area.

As mentioned above, depressive disorders, both treatment responsive and resistant, can include significant social, emotional, or somatic dysregulation. Dysregulation can occur though hypo or hyper arousal; both of which include somatic components (such as breath, touch, temperature, etc.) which maybe unique to each person. The aspect of self-regulation being focussed on in this study is emotional self-regulation. Emotional self-regulation is the body’s way to seek stability. It is a process where one can manage feelings and maintain hope amid stressful situations (de Almondes et al., 2021). Currently, no dance/movement therapy technique has been identified to aid in this process of self-regulation in treatment resistant depression. Fundamentally, dance/movement therapy uses the body's power in deepening both the exploration and expression of the mind. Due to this fundamental use of movement, it is imperative to look at the impact of movement on the brain, specifically on the sympathetic nervous system. Understanding the nervous system and the homeostatic changes associated with it is a critical first step in determining a treatment plan to address anxiety, stress, and emotional disorders such as treatment-resistant depression (Jerath et al., 2015). Along with changes in the autonomic nervous system, emotions also bring various bodily reactions, such as feeling stiff, shortness of breath, tension headaches, sensation of pins and needles, etc. In this way, small or large movement shifts in our overall body or specific postures are indicators of different emotional states and the activation of the autonomic nervous system. These movement can safely occur within kinesthetic empathy.

As mentioned above, depressive disorders, both treatment responsive and resistant, can include significant social, emotional or somatic dysregulation. A wide range of complexities arise due to this. They include inflammation, unspecified emotional distress, stress and obesity (Bakal et al., 2008; Beurel et al., 2020; van Strien, 2018). According to Kovacs et al. (2008), one’s neurobiological infrastructure plays a key role in regulation, specifically in processing and understanding sadness. Both responsive and resistant depression are often co-morbid with anxiety, thus only further complicating the series of symptoms needing to be managed (Hirschfeld, 2001; Pericleous, 2011; Zhiguo & Yiru, 2014). Holmes et al. (2006) speak to the presence of co-morbidities, community attitudes, demographic factors, and constraints in social support as factors that may obscure the treatment path in resistant mental illnesses. This distressing cluster of symptoms not only diminishes patients’ quality of life but also increases the financial burden on them (Olchanski et al., 2013). Patients often go from one doctor to another in search of suitable treatment for such varied comorbidities, only further increasing stress and ultimately causing dissatisfaction in treatment (Trevino et al., 2014). Recurrent exposure to such distress keeps the sympathetic nervous system (SNS) charged and activated (Won & Kim, 2016). When activated, the SNS responds by commanding the body to flee, fight, or even freeze (Roelofs, 2017). In all three states, the body experiences dysregulation and may require vagarious co-regulation and rehearsal of self-regulation interventions to attain a state of homeostasis or ease. Factoring-in the lived experience of stress in TRD with the relational with the sensory aspects of kinesthetic empathy in dance/movement therapy; the rationale for utilizing sensory intelligence in self-regulation is made apparent. The authors propose that kinesthetic empathy maybe one of the ways in which clients can learn to better comprehend somatic cues, practice somatic intelligence and cope in difficult environments.

### Dance/Movement Therapy as Treatment for Depression

As an art form, Coubard et al. (2011) identified the benefits of dance to focus on prevention rather than remedy of illnesses. This is also consistent in current dance/movement therapy literature (Koch et al., 2019a). Research suggests that dance/movement therapy can be an effective adjunctive tool to traditional approaches by reducing symptom related distress and, subsequently, increasing overall treatment satisfaction (Chase, 1953; Haller et al., 2019; Laws & Conway, 2019). Main factors contributing to this include: incorporation of structured dance and music as ways of self-expression and coping (Tavormina & Tavormina, 2018), enhancing socialization via participation in group therapy sessions (Hagensen, 2015), promoting vitality (Levy, 1988; Meekums et al., 2015a), and placing emphasis on therapeutic movement relationship between the dance/movement therapist/s and client/s (Meekums et al., 2015a). Of these factors, the therapeutic movement relationship is unique to the practice of dance/movement therapy.

Young (2017) describes therapeutic movement relationship as “a shared presence of body, mind, and spirit” between the therapist and client. She places further emphasis on safety and collaboration as essential components that create “resonance in the relationship” (p.104.). She explains therapeutic movement relationship as a concept rooted in kinesthetic attunement, which in-turn occurs due to kinesthetic empathy. Thus reinforcing a conceivable connection between kinesthetic empathy and the therapeutic movement relationship in dance/movement therapy.

Two systematic reviews have been conducted on dance/movement therapy and depression (Karkou et al., 2019; Meekums et al., 2015a). The review by Meekums et al. (2015b) includes a total of three studies, with 147 participants (107 adults and 40 adolescents). The review conducted by Karkou et al. (2019) includes eight studies through a qualitative narrative synthesis with 351 participants (192 dance/movement therapy participants and 159 control participants (Karkou et al., 2019, p. 10). Similarities were evident in the dance/movement therapy intervention used across both reviews (i.e., all 11 individual studies). They both actively used the body as a resource to increase awareness, reclaim individual stories (through use of metaphors), process narratives of trauma from a somatic lens, foster self regulation, address negative self-evaluation, and promote an overall boost in mood. Despite the overall findings of the review by Meekums et al. (2015b), where the effectiveness of dance/movement therapy as a primary depression treatment intervention is inconclusive, in a more recent review by Karkou et al. (2019) they suggest that secondary symptoms of depression, such as self-esteem, quality of life and socio-occupational functioning, could be alleviated via active participation in dance/movement therapy sessions. However, it is unknown if this conclusion can be applied to various populations, ages, and cultural groups. Additionally, current literature does not adequately focus on understanding the long-term effects of dance/movement therapy in depression treatment, including the studies mentioned above. The nacency of the dance/movement therapy field, along with limited evidence-based literature on dance/movement therapy as a treatment for depression, poses a strong challenge for its intentional use with clients. With respect to the focal population of this study (individuals living with treatment resistant depression), there is currently no published research on the effects of dance/movement therapy as a treatment intervention in resistant depression. This scarcity of literature has also been briefly discussed in the paragraph below.

### Gap in Research

At present, the pool of dance/movement therapy literature more broadly faces two main difficulties. Some studies focus on dance while acknowledging dance/movement therapy theories in their literature reviews. For example, the use of OULA®, a dance fitness program in depression treatment (Hellem et al., 2020), the use of dance activations like Rumba, Vals and Aerobics in depression treatment (Akandere & Demir, 2011) and, the use of ballroom & contemporary dance in recovery chronic illnesses including chronic depressive symptoms (Bruyneel, 2019). These studies make make rich contributions to understanding the effect of dance in depression treatment and the use of dance as an intervention is supported by dance therapy theory. However, the lack of a clear, reliable dance therapy intervention led by a trained dance therapist limits them from being considered in the pool of dance therapy literature in depression treatment.

These two factors drastically limit the research that becomes applicable in treatment. With regards to TRD, there is an even loftier gap in dance/movement therapy research. Currently no published article or dance/movement therapy intervention has been identified to specifically address dysregulation in adults with TRD.

### Relevance of Study Participants

The country of clinical practice of each participant is relevant within the larger socio-cultural dynamics of the field of dance/movement therapy. With dance/movement therapists actively moving towards operating from a space of social and restorative justice, the implicit assumption that dance/movement therapy outside of Eurocentric cultures is primarily practiced solely as part of the social milieu or integrated with other magico-religious and spiritual practices needs to be questioned. Although this was not the intended aim of this study, the participant demographics of this study contributes towards de-centring master’s level trained dance/movement therapists as belonging to and practicing in Eurocentric cultures. The findings also provide formative data on the clinical use of dance/movement therapy in treatment of resistant depression in India, Philippines, and Barbados. The sample of this study did not affect the methods of data analysis used in this study.

## Methodology

Understanding depression as an illness of impaired regulation through different theories is still to be fully grasped (Joormann & Gotlib, 2010, p. 281). Yet, evidence propels us to explore the feasibility of symptoms reduction and overall depression treatment from a lens of regulation (Compare et al., 2014; Kong et al., 2019; Sloan et al., 2017). This survey study was designed to gather data to help answer two specific research questions:

(i) How is kinesthetic empathy used in dance/movement therapy sessions with people living with treatment-resistant depression?
(ii) What (if any) are the key factor/s in supporting the link between kinesthetic empathy and self-regulation within dance/movement therapy?

### Aim

The survey study aimed to understand how kinesthetic empathy is used to foster self- regulation in adults living with treatment-resistant depression by formally trained dance/movement therapists across the world.

### Design

Data was obtained from surveys conducted online with formally trained dance/movement therapist. The survey included eleven questions, seven of which were closed and four of which were open-ended questions. The first seven questions ensured that interested dance/movement therapists were eligible to participate in the study. The next four questions were designed to gain information on the use of kinesthetic empathy. The survey was built in Qualtrics and circulated via a Qualtrics link.

### Ethics

This study was approved by The University of Melbourne’s Human Rights and Ethics Committee (HREC) in August 2021. Approval from the Human Research Ethics Committee (HREC) ensured that the study guidelines and practices complied with the National Statement on Ethical Conduct in Human Research. A copy of the informed consent, data storage plan, distress protocol, recruitment advertisement, and plain language statement were submitted and approved in support of this study.

### Participants

The participants of this study were master’s level trained dance/movement therapists from anywhere in the world. Study advertisements were circulated via member directories of various dance/movement therapy associations. Informed consent was obtained from all participants included in this study. No identifying information is presented in this article.

### Inclusion Criteria

The eligibility criteria for this study includes clinicians who: (i) Hold a master’s degree in dance/movement therapy; (ii) Have worked for a minimum of two years in the field of dance/movement therapy. There was no specific setting requirement. Settings can vary from in-patient psychiatry, long term clinical settings, private practice, etc.; (iii) Have clinical experience with adult clients living with treatment-resistant depression for at least six months (overall); (iv) Have used/use kinesthetic empathy to foster self-regulation in clients with treatment-resistant depression.

### Exclusion Criteria

The exclusion criteria for this study includes (i) Individuals who consider themselves to be dance/movement therapist but do not have formal masters level training in dance/movement therapy, and (ii) Dance/movement therapists who have formal masters level training but do not either 2 year of work experience or have not worked with individuals living with treatment-resistant depression for at least six months (overall).

### Recruitment Strategy

Considering COVID-19 protocols during the planning and recruitment stages of this study, advertisements and correspondence were only carried out electronically. The invitation to participate was shared with potential participants by via newsletters and monthly emailers of various professional dance/movement therapy associations across the globe. Since this study is aimed to understand the use of kinesthetic empathy by trained dance/movement therapists, recruitment via dance/movement therapy associations was a viable way to seek participants representation of the population in this study. Additionally, the survey was designed to ensure eligibility of each therapist who participated. The email advertisement clearly indicated the eligibility criteria, and expectations of participants.

### Data Collection and Analysis

The full survey is presented below (Supplementary file 1). Since the first seven questions were screening questions to confirm eligibility, these questions were not analysed categorically. However, the four open-ended questions were grouped into four categories. The rationale for this type of analysis is in line with the study aim to learn from dance/movement therapists’ experiences. The findings of this study contribute knowledge on the nature in which kinesthetic empathy is used with a specific population and is not intended to make clinical generalizations.

### Data Storage

Once the surveys were completed on Qualtrics, the responses were alphabetically coded based on random letters and stored securely on Media Flux (and deleted from Qualtrics). Participants were informed of this in a plain language statement provided via email at the time of recruitment.

## Results

### Participant Characteristics

The recruitment advertisement and an invitation to participate was sent to 140 dance/movement therapists via email and shared with 135 members of a local dance/movement therapy association (Melbourne, Australia) in their monthly newsletter for November 2021. Of the 275 dance/movement therapists who received information about this study, 13 commenced the survey. One participant attempted to complete the survey twice but was not eligible based on the inclusion criteria. Three other dance/movement therapists stopped completing the survey midway. Upon correspondence with them, they shared their inability to complete the survey due to being overworked and busy. From the 13 surveys commenced, five were not included in the analysis (one duplicate, one ineligible and three incomplete). Eight survey responses from India (n = 3), Barbados (n = 1), Portugal (n = 1), the Philippines (n = 1), and the USA (n = 2) were included in the data analysis.

Overall, the survey received a 4.72% response rate. Possible causes for this are difficult to name. However, some speculations include highly demanding work hours from therapists during COVID-19, professional exhaustion and/or lack of using kinesthetic empathy as an intervention for treatment-resistant depression. The first response was obtained in November 2021 and last response was obtained in March 2022. Although the sample is small, it provides introductory information that can be built upon in both, dance/movement therapy and allied healthcare research. This study is unique as data has not been obtained solely from countries like Europe and the United States, where most of the research in TRD has been previously conducted.

### Summary of Results Obtained

Four open ended questions were asked in the survey. The results of which have been outlined below and discussed in the next section.

1. The first narrative question stated, *could you mention any specific ways in which you used kinesthetic empathy in your work?* Four participants alluded to interoception, three mentioned rapport development and two participants mentioned using kinesthetic empathy as a pathway to regulation.
2. The second narrative question stated, *in your experience, how do clients respond to kinesthetic empathy in dance/movement therapy sessions?* Four participants named observable responses and four participants named perceived client-felt emotional responses to kinesthetic empathy in dance/movement therapy sessions.
3. The third narrative question stated, *please specify how you are able to attribute any mentioned changes to kinesthetic empathy?* Five participants mentioned client self-report to attribute observed and emotionally felt responses to kinesthetic empathy. Three survey participants mentioned clinician’s observation of movement pathways in their clients as a plausible link between the observed changes noticed by therapists and kinesthetic empathy.
4. The fourth narrative question stated, *based on your experience, are there any key factor/s that support the potential link between kinesthetic empathy and self-regulation?* Four survey participants mentioned co-regulation and four survey participants mentioned increased attunement with self as potential links between kinesthetic empathy and self-regulation within dance/movement therapy sessions.

The narrative responses have been discussed in four categories in the Discussion section below.

## Discussion

### Narrative Responses

#### Category 1: Rationale for Using Kinesthetic Empathy in Dance/Movement Therapy

**Fig. 1:**
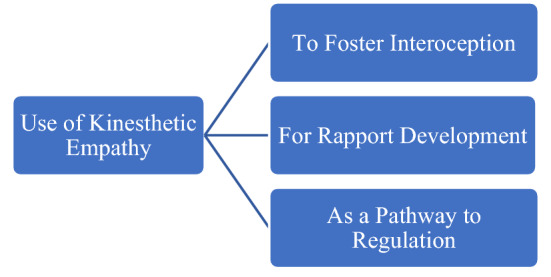
Use of kinesthetic empathy

The responses obtained suggest that kinesthetic empathy was actively used by dance/movement therapists with the rationale to foster interoception, for rapport development, and as a pathway to regulation in dance/movement therapy sessions with adults living with treatment resistant depression (as seen in Fig. 1). These three uses have been discussed below.

##### To Foster Interoception

Interoception is the process of gathering information through sensory experiences (Craig, 2003). As described by Marshall et al. (2022), “The human body is the seat of our self-awareness” and relies on the process of interoception to identify and express emotions (p. 1). Four of the eight survey participants alluded to this process in their answer to the question, ‘how do you use kinesthetic empathy in your dance/movement therapy practice with people living with treatment resistant depression?’ The phrases, “to gather information” and, “to better understand” were used in the responses. Participants also touched upon the organic nature of interoception to better understand another’s emotional state. This is validated by the anatomical functioning of interoception which has neuroscientific evidence linking it to emotional processing and to development of a self-concept (Craig, 2008; Hindi, 2012).

Until the first decade of the twenty-first century, interoception was understood merely as a physiological function; one that mainly included activation of the fascia (or connecting tissues) and caused muscular movements (Ceunen et al., 2016). However, with the increased neuroscientific studies on interoception, Chen et al. (2021) discovered that interoception not only activates the connecting tissues in the human body but also activates the unmyelinated nerve endings of the brain related to emotions. This finding is particularly helpful as depression is considered to be an interoceptive disorder (Paulus & Stein, 2010). Particularly, moderate/severe depression has been found to be inversely proportional to interoceptive indicators like body listening, body noticing, and emotional awareness (Dunne et al., 2021). Since sensory experiences have emotional, motivational, and behavioural aspects which are directly related to homeostasis (Schleip et al. (2012); tapping into the power of interoception in depression treatment seems justified and it’s use is corroborated in the survey responses.

The value of interoception in contributing to a stronger self-concept was also found in the responses to this first survey question. One of the participants described using kinesthetic empathy to understand the “embodied culture” of their client. This is a key finding as it illuminates the use of kinesthetic empathy in a relational way and to understand clients’ lived experiences. Likewise, two other participants reinforced the use of kinesthetic empathy to deepen their understanding of their clients’ “whole self” and “inner experiences”. Some examples of interoception mentioned by the participants include observation of posture, facial expression, gesture, and muscle tension. Hindi (2012) also notes muscle tension to be an example of interoception.

An aspect of interoception that maybe beneficial to depression treatment is the process of tracking. Tracking is one of the five step sequential interventions to optimize the use of interoception in dance/movement therapy sessions as proposed by Hindi (2012); tracking is a process that engages embodied awareness, by identifying and tracking physical sensations (p. 136). It is not an action-oriented state that demands more from clients but instead, creates a space for being attuned with oneself. In relation to treating resistant depression, one pilot study on understanding the effects of deep brain stimulation on interoceptive processing has been conducted (Waters et al., 2020); the results show that deep brain stimulation affected interoceptive processing in the cortex however no statistical evidence of change in depressive symptoms was found. (Waters et al., 2020) note the need for further research on the possible benefits of interoception in depression treatment this topic. Similarly, the results of this survey study also support this.

##### For Rapport Development

Three survey participants highlight using kinesthetic empathy to develop a rapport and “meet clients where they are at”. Meeting clients “where they are at” is a common dance/movement therapy phrase rooted in Chace’s (1993) philosophy of dance/movement therapy. Chace believed in the power of rhythmic movement to support non-verbal expression or use ‘dance for communication’(Chace et al., 1993). Similarly, one of the survey participants pointed to using kinesthetic empathy to help their clients non-verbally communicate with one another. These three participants described using kinesthetic empathy to ultimately engage better and more actively with clients.

As part of the rapport development, participants also highlighted kinesthetic empathy’s foundational role in building a therapeutic alliance with their clients. Aligning with current literature (Crooks & Mensinga, 2021; Fischman, 2009; Young, 2017), this finding is valuable in identifying the continued benefits of kinesthetic empathy in the specific context of dance/movement therapy with people with resistant depression. Two participants emphasised the importance of kinesthetic empathy in maintaining a rapport through the duration of the therapy process. One survey participant noted using kinesthetic empathy specifically in the “warm-up stages” and towards the “end of client’s therapy journey to offer grounding”. Other survey participants described it as,’ “the core of their work” and, “integral in all phases of dance/movement therapy”; the rationale for which was the importance of kinesthetic empathy in “devising appropriate and client centred interventions in therapy”.

Three specific ways of using kinesthetic empathy for rapport development were noted: to create a safe space, mirror, and attune with clients. Embodied attunement in dance/movement therapy is a shared emotional experience of, “connecting with patients on an empathic level that reaches deeper than verbal connection” (Lacson, 2020, p. 8). One of the participants attributed reaching a “continuous and consistent state of kinesthetic empathy with long term clients” to attunement and moving with clients. The authors believe that the therapist’s attunement with their client may help deepen the therapeutic alliance. This potential co-relation between the two is rooted in mutual trust, appropriate self-disclosure, communicative intentions and has been widely cited in scholarly literature in attachment-related psychotherapy and interpersonal communication research (Erskine, 1998; González et al., 2019; Talia et al., 2019). Likewise, one of the participants directly spoke to using kinesthetic empathy as it plays an “integral role in fostering relational connections” between the client and the therapist.

##### A Pathway to Regulation

Two therapists attributed their use of kinesthetic empathy as a pathway to regulation in people living with treatment resistant depression. One of the survey participants said, “Connecting via kinesthetic empathy, helps me attune to others, which in turn lends itself beautifully to the process of co-regulation”. Another participant described using kinesthetic empathy to offer grounding and self-regulation. Although this is a small sample to ascribe emotional regulation to kinesthetic empathy; it is pioneering data in the specific population of treatment resistant depression.

The experiential knowledge of co-regulation shared by dance/movement therapists is corroborated by a developmental understanding of emotional regulation within family systems. Murray and Rosanbalm (2017) identify three aspects of co-regulation (a) providing a warm, responsive relationships, (b) structure and (c) teaching/coaching self-regulation skills. The first and third aspects have been also identified through these survey study results.

Although the direct causal factors to link kinesthetic empathy and emotional regulation remain ambiguous. The survey results suggest a safe holding environment (Winnicott, 1960) and movement within the therapeutic alliance as a possible link between kinesthetic empathy and emotional regulation. One of the participants directly spoke to “safety” as an important factor in their use of kinesthetic empathy. The authors believe that expressive movement within a safe space creates the opportunity for emotional self-regulation. Imus (2021) aptly uses the term, ‘creative courage’ to describe the result of experiencing safety and trust in therapy. Since ‘creative courage’, is rooted in trust and safety which, are in-turn rooted in kinesthetic empathy, creative courage may also be understood as a mechanism of change in learning or rehearsing ways to emotionally self-regulate (Imus, 2021). Another significant aspect of creating safety in dance/movement therapy sessions is through mirroring. Mirroring can be understood as a process that

“occurs when two people make similar body movements that are coordinated or slightly echoed in time. The therapist may echo the exact movements of a client, or may imitate the quality of the movement; for example, if a client is moving with a slumped posture, the therapist may adopt these movement qualities as well”

(McGarry & Russo, 2011, p. 178). It is through the process of mirroring that a therapist is offered a window into their client's lived emotional experience, thus fostering and deepening empathy within the therapeutic alliance.

Based on neuroscientific data, the right brain hemisphere is home to affect recognision and processing. According to Lacson (2020) when emotional processing is facilitated by engaging the body and it’s vitality, it can enable rewiring of neural pathways towards emotional regulation (p. 164). Vitality is an essential aspect of dance/movement therapy and has been identified as such across human culture by both by both formally trained dance/movement therapists and by native American people groups participating in cultural healing dance practices in the United States (Barnstaple, 2016). Vitality in dance/movement therapy is commonly seen in its potential to offer joyful or cathartic experiences to clients (Karkou et al., 2019; Koch et al., 2019b; Lauffenburger, 2020; Tillberg, 2012). It should be noted that vitality is the symptomatic opposite to depressive symptoms of low mood and thus maybe highly beneficial with this population.

#### Category 2: Therapist-Described Client responses to Kinesthetic Empathy

**Fig. 2:**
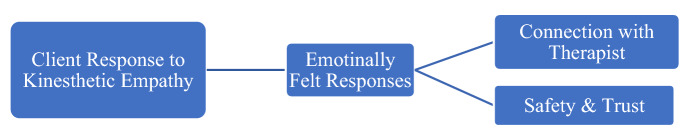
Client response to kinesthetic empathy

The results suggests that survey participants’ client responses to kinesthetic empathy can be categorised in two broad categories, that is, observable, and emotionally felt responses (Fig. 2).

##### Observable Responses

Five participants named observable responses to kinesthetic empathy in dance/movement therapy sessions. These included increased eye contact and touch, exploring movements, use of metaphors, verbalization of body sensations during movement, engaging in observing, following, and mirroring of large movements in group settings, ease in embodiment. Like kinesthetic empathy, mirroring is also not unique only to the process of dance/movement therapy and can also occur in the social milieu. The process of mirroring is rooted in affective neuroscience and the mirror neuron network (MNS), this system enables individuals to view and embody another’s facial expressions, posture, and muscular patterns which can also influence and illicit a visceral feeling in the person. The survey responses received did not include specific examples of the “exploring movement” and “ease in embodiment”, however, a common way to explore movement is to experience different effort qualities, in different levels of space in the kinesphere (Bartenieff & Lewis, 2013).

One of the participants, distinguished their client’s response to kinesthetic empathy based on the type of setting (i.e., group vs individual). Reinforcing the results from the previous question, participation in “mirroring and shared rhythm” were named as common ways of responding to kinesthetic empathy in groups. Alternatively, “active engagement in subtle and gentle movements” was noted as a more common response to kinesthetic empathy in individual sessions.

These observable responses provide imperative information that can be used towards creating measurable tools within dance/movement therapy. However, a challenge with this data set is the difficulty of attributing these observable responses purely to kinesthetic empathy in dance/movement therapy sessions.

##### Emotionally Felt Responses

Four participants described they perceived client-felt emotional responses to kinesthetic empathy. These included an increase in trust, sense of safety, and connection. Emotionally felt responses may not be easily noticed or observed by all. Instead, a strong therapeutic presence is needed to monitor and note these emotional nuances as a response to kinesthetic empathy.

Like the distinguishing factor of group versus individual setting mentioned earlier; a participant divided their perception of client responses in terms of conscious and unconscious states of mind. They identified an “increase in responsiveness” to a conscious state and a “welcoming attitude” to an unconscious state of mind. One survey participant particularly validated an “increase in comfort and safety which ultimately strengthened the therapeutic relationship and reduced emotional resistances” within the session.

Two specific sub-themes emerged under emotionally felt responses:

(i) *Connection with therapist*: Survey responses suggest an increase in connection with the therapist to be a distinct response to kinesthetic empathy. Mirroring is one of the ways in which this connection was felt. This aligns with Rova (2017)’s identification of mirroring as one of three thematic developments of kinesthetic empathy.
(ii) *Safety and trust*: In the context of treatment resistant depression, studies highlighting the importance of safety and trust within therapeutic movement relationship (Young, 2017) are missing. While there are studies on evaluating the safety of drugs in treatment resistant depression (Al-Harbi, 2012b; Karp et al., 2014; Palhano-Fontes et al., 2019), little is known regarding the specific potential of emotional safety and trust in treating resistant depression through dance/movement therapy. The results of this survey point to clients’ essential need to feel emotionally safe. One of the participants emphasized the importance of kinesthetic empathy by stating, “I cannot imagine setting up a working alliance, rapport or trust without kinesthetic empathy”. Another participant added, “In my years of experience as a dance/movement therapist and working mostly with groups, I have found patients to be especially responsive to the concept of kinesthetic empathy. I often will spend time speaking about it and teaching them to develop their own sense of kinesthetic empathy.” From this response, it can be ascertained that clients’ gaining insight into their own embodied experience or kinesthetic abilities may also be an emotionally felt response to kinesthetic empathy.

Embodied experiences are those in which individuals are not only intellectually engaged but are also involving the different senses of their bodies to understand and make meaning of their environment and interactions, Johnson (2015) suggests that embodied experiences not only include, physical experiences (like touch, body posture) but also include interpersonal (like felt sense of comfort) and larger collective cultural experiences. Likewise, kinesthetic abilities are skills rooted in kinesthetic intelligence. Kinesthetic intelligence is one of six types of intelligence according to Howard Gardner’s Theory of Multiple Intelligence (Gardner, 2011). Kinesthetic intelligence uses the body to problem solve in a given situation through keen sensory perception, dexterity, balance, a robust body-mind connection, and/or bodily expressiveness (Gardner, 2011; Singh et al., 2017). Similar to the survey participant’s response about ‘teaching kinesthetic empathy’ to clients, an earlier study validates the increase in recognizing one’s kinesthetic abilities with an increase in taught empathy (Federman, 2011). Based on these survey results, experiencing safety and trust within the therapeutic alliance can contribute to learning and living in a more embodied manner.

#### Category 3: Changes in self-regulation pattern in clients

**Fig. 3:**
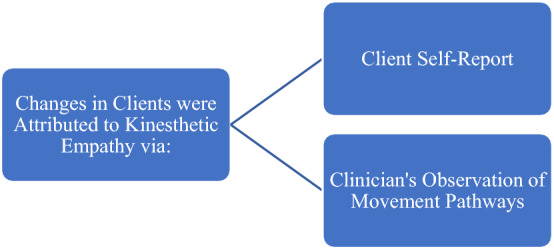
Attributing changes in patterns of self-regulation to kinesthetic empathy

The survey results indicate that dance/movement therapists attribute changes in self-regulation to intervention of kinesthetic empathy in two main ways, that is via the (Fig. 3):

##### Client Self-report

Given that mental health struggles are subjective, self-report has been an invaluable way to understand client experiences in mental health treatment. Five survey participants mentioned self-report to attribute the above-mentioned responses (observed + emotionally felt) from their clients to kinesthetic empathy.

The survey results indicate the nature of self-report to primarily include, “verbal sharing”, which was identified to relate with “emotional release”. In two instances, the verbal sharing of the clients provided the therapist with “insight” into the client’s experience. One of the survey participants said, “I learn that the patient has found the process of kinesthetic empathy to be beneficial … or to have contributed to some shift within them.” Another participant reinforced this by stating “insight and reflection within the therapy space” as possible due to kinesthetic empathy.

##### Clinician’s Observation of Movement Pathways

**Fig. 4:**
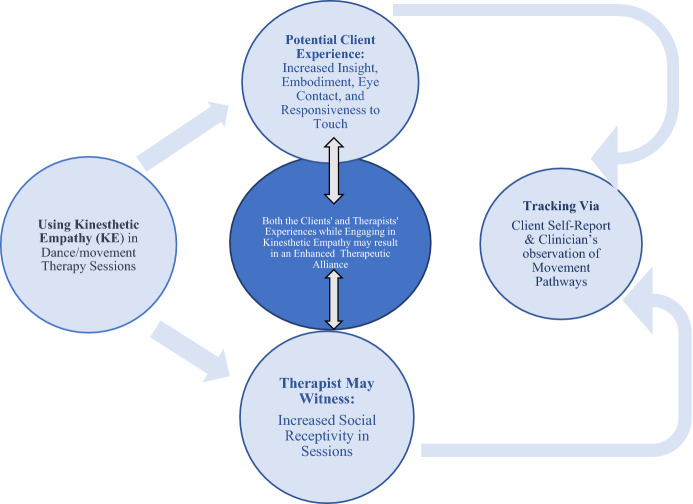
Visual representation of the cycle of change and tracking while using kinesthetic empathy in dance/movement therapy session

Three survey participants mentioned observable movement pathways in their clients as a plausible link between the observed changes noticed by therapists and kinesthetic empathy. This was described as “increased movement pathways across space and their kinesphere expanded after experiencing authentic embodied empathy.” One of the survey participants noticed “observable body postures and movement patterns shifts”. Specially eye contact and “smiling” while moving were also named by another participant. Participants also named observing movement pathways when clients didn’t seem “as cautious as they did when we first began”. From a social psychology lens, these non-verbal responses may be considered as indicators of a willingness to socially engage. Hence, the observed pathways have been termed as ‘increased social receptivity in sessions’ in Fig. 4 below. Movement pathways were also observed when clients didn’t seem “as cautious as they did when we first began”.

Hence, based on the survey findings, kinesthetic empathy may result in changes for both the client and the therapist, which may have a beneficial effect on the therapeutic alliance. The following is a visual representation of the patterns of change and tracking of (any) progress (Fig. 4).

#### Category 4: Potential Links Between Kinesthetic Empathy and Self-regulation

**Fig. 5:**
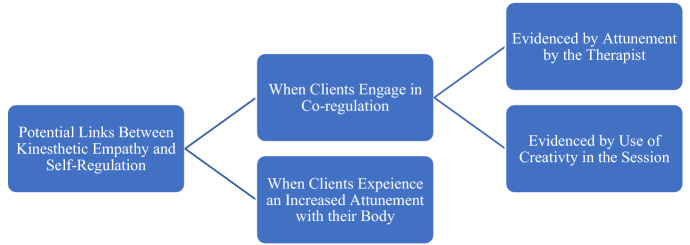
Potential links between kinesthetic empathy and self-regulation

The responses obtained suggest the potential link between kinesthetic empathy and self-regulation to mainly be, co-regulation between the therapist and client and increased client attunement with self (see Fig. 5).

##### Co-regulation

Four survey participants mentioned the occurrence of co-regulation as the potential link between kinesthetic empathy and self-regulation in dance/movement therapy sessions. Survey participants alluded to process of self-regulation as marked by active engagement in co-relation between the client and therapist. One participant said, “co-regulation seems to be key in being able to link kinesthetic empathy and self-regulation”. Mindfulness and being in the “here and now” were also noted as a potential link. The process of teaching, learning, and practicing self-regulation within the safety and familiarity of the therapeutic alliance was also emphasized. One of the responses indicated the long duration of time that this process can take; they used the phrase, “after many months” in the context of kinesthetic empathy fostering self-regulation. Based on the answers, the process of co-regulation was associated with the following:

(i) *Attunement from therapist*: Closely linked to co-regulation is attunement. three participants highlighted the benefit of the therapist’s attunement to the client. They said attunement helps “better offer movement interventions designed to support or initiate self-regulation or co-regulation” and fostering a feeling of being connected with the therapist, “being seen/matched which creates a stronger bond/alliance between in the partnership”.
(ii) *Creativity within the session*: Risk taking and creativity within the session and active engagement in non-verbal expression was also closely associated with being able to self-regulate over time.

##### Increased Attunement with the Body

Four survey participants mentioned increased attunement with self for clients as a potential link between kinesthetic empathy and self-regulation within dance/movement therapy sessions. One participant described this process of increased attunement with clients as a gentle unfolding of movement coinciding with mirroring. An increased use of the body was reinforced in another participant’s response, where they described, “deeper, embodied, meaningful and authentic connection with the body” and how understanding the meaning of their verbal expression paves the way for regulation. Another participant felt that the key factor in the link between kinesthetic empathy and self-regulation was, “understanding and engaging with the client's unique body presentation and movement language to understand what self-regulation means in their context.” Although adequate evidence on the link between increased attunement with one’s own body and one’s increased emotional capacity is not yet available, the authors suggest this possible link based on previous literature describing the positive benefits of symbolic play in emotional development and regulation (Wieder, 2017). Concurrently, Greenspan and Wieder (1998) attributed the positive benefits of engaging in symbolic activities (like dance or play) to the diverse, unique meanings that could be derived from symbols. Further, they identified symbolic engagement to help individuals separate reality from fantasy, which eventually assisted in self-regulation.

### Therapeutic Factors

To better understanding the nature of kinesthetic empathy within treatment resistant depression, results were examined based on the therapeutic factors and mechanisms of change in creative arts therapies as outlined by De Witte et al. (2021). De Witte et al. (2021) emphasize the importance of conceptualising change practice research to understand the “factors that lead to therapeutic change” (p. 2). Based on the survey responses, kinesthetic empathy can be associated with both dance/movement therapy specific factors and mixed-type factors. These have been identified and tabulated in Tables 1 and 2 below.

**Table 1:** Categorizing kinesthetic empathy into therapeutic factor of change

Use of kinesthetic empathy		Type of therapeutic factor of change (2012)
Aim	Mechanism	
To foster interoception	Via sensory awareness	DMT-SF*
For rapport development	Via embodied attunement	CAT-JF**
A pathway to regulation	Via emotional processing though active body engagement	Common factors of psychotherapy from DMT studies

**Table 2:** Categorizing client responses to kinesthetic empathy

Client responses to kinesthetic empathy	Type of therapeutic factor of change (2012)
Observable client responses to kinesthetic empathy	
Touch	Not specified
Exploring movement	Not specified
Ease in embodiment	DMT-SF
Use of metaphors	CAT- JF
Verbalization of body sensations	DMT-SF
Engaging in mirroring	DMT-SF
Emotionally felt responses to kinesthetic empathy	
Strengthening the therapeutic alliance (increase in connection, safety, and trust)	MF***

*DMT-SF Specific therapeutic factors related to dance/movement therapy. **CAT-JF Joint therapeutic factors shared by the creative arts therapies. ***MF therapeutic factors mixed factors from creative arts therapies and psychotherapies

*Key* (De Witte et al., 2021)

### Recommendations for Clinical Practice

The following table presents formative movement interventions that may be incorporated by dance/movement therapists while working adults living with treatment resistant depression based on survey results (Table 3).

**Table 3:** Movement intervention recommendations for use in dance/movement therapy practice

*Population* Adults living with treatment resistant depression				
Potential goals	Movement recommendation	Type of setting		Tracking
		Groups	Individual	
Co-regulation	(1) Mirroring	✓	✓	Information from client self-report and clinician’s observation of movement pathway is recommended to track the potential benefits of kinesthetic empathy
	(2) Sharing rhythms	✓	✓	
	(3) Use of metaphors	✓	✓	
	(4) Exploring movement	✓	✓	
Attunement with self	(5) Verbal discussion about developing one’s own sense of kinesthetic empathy	✓	✓	

### Study Limitations

This study has a few acknowledged limitations.

(i) The semantics of question 10, which is framed as: “Could you mention any specific ways in which you used kinesthetic empathy in your work?” suggests kinesthetic empathy to primarily be an “intervention” which is “used” in dance/movement therapy. Data on if or how this language impacted survey participants is unknown.
(ii) While a strength of this study is that it includes perspectives of therapists-of-colour, the small sample size in this study does not allow for generalization of results across cultures.
(iii) The recruitment strategy was designed to be a representative of the larger participant population. However, due to the low response rate, these findings do not provide adequate information to be representative of the participant population. Additionally, since the recruitment occurred during the COVID-19 pandemic, if and how environmental factors like contracting COVID-19, fear of infection, isolation and/or grief experienced by each participant maybe considered an independent variable is undetermined. This is relevant as the survey responses were received from geographical location with high infection and transmission rates during data collection. Future surveys that report on symptoms reduction and client responses based on the formative recommendations for dance/movement therapy practice mentioned above is encouraged.

## Conclusion

This paper presents the results of a survey study examining dance/movement therapists’ use of kinesthetic empathy when working with people living with treatment-resistant depression (TRD). The aim of the survey was to collect data on if and how kinesthetic empathy is used to foster self-regulation in adults living with TRD by formally trained dance/movement therapists across the world. Based on the findings, kinesthetic empathy was similarly described by all participants as a core element of dance/movement therapy practice with this population. The importance of kinesthetic empathy was noted in connecting and attuning with the client, creating safety, and initiating co-regulation within the session. The longitudinal use of kinesthetic empathy throughout therapy was also highlighted. Participants mentioned using kinesthetic empathy to both initiate rapport development and foster/ actively engage in the therapeutic alliance across the duration of therapy.

While the four categories of data obtained from this study, provide practice-based insights on the use of kinesthetic empathy to foster self-regulation in dance/movement therapy, the findings of this study also shed light on discrepancies and differences of opinion on kinesthetic empathy as an intervention. It should be noted that universal agreement on whether kinesthetic empathy is a concept, principal or element in dance/movement therapy is yet to be attained both in the larger field of dance/movement therapy as well as in this sample. For example, even within this small sample, the diversity of responses and words used to describe kinesthetic empathy include, ‘concept’, ‘mechanism of change’ and ‘process’. Irrespective of this, the centrality of kinesthetic empathy in dance/movement therapy sessions was clear in these findings. However, a collective decision to either converge or openly accept divergent views on kinesthetic empathy should be addressed in the field. Special training and emphasis on the separation between the performative and relational aspects of kinaesthetic empathy should be focused on in teaching and training spaces. This is especially important as most dance/movement therapists also have a background of performance, thus opening them up to experience kinesthetic empathy in more than one way or contexts.

Although kinesthetic empathy is positioned as an intervention in this survey, the study findings suggest that kinesthetic empathy is experienced by both the therapist and client and is hence maybe a relational, participatory intervention rather than a more conventional and directive intervention. The visual representation of the patterns of change and tracking (Fig. 4) within dance/movement therapy propels researchers to also study how intentional use of kinesthetic empathy may be a successful adjunctive tool in overall treatment by keeping clients motivated and engaged in their long treatment plans. Since dissatisfaction with treatment is a common demotivating factor that can also exacerbate symptoms of TRD, utilizing kinesthetic empathy can be envisioned as an ‘enhancer’ to the treatment process and client experience.

The need for tracking symptoms or change in patterns was also reinforced by this survey study. As kinesthetic empathy is a novel intervention in healthcare, it is essential for therapists to create more standardised ways of capturing the effects of kinesthetic empathy so that it may be used to advocate for dance/movement therapy in healthcare. This is even more necessary for vulnerable populations, such as individuals living with treatment-resistant depression. The survey results also suggest that future research on unearthing the use and impact of kinesthetic empathy may be guided by the relational process factors type of Change Practice Research (Elliott, 2011).

## Supplementary Information

Below is the link to the electronic supplementary material.

Supplementary file 1 (PDF 132 KB)
