# Supplementary material for: The Use of Kinesthetic Empathy with Adults Living with Treatment Resistant Depression: A Survey Study
Source: Am J Dance Ther. 2022 Sep 23;44(2):115–42. doi: 10.1007/s10465-022-09371-4 (PMC9510258; doi:10.1007/s10465-022-09371-4)
Supplement: Supplementary file 1 — (PDF 132 KB) [file 10465_2022_9371_MOESM1_ESM.pdf]

Supplementary Document 1

Manuscript Title: The Use of Kinesthetic Empathy with Adults Living with Treatment Resistant Depression: A Survey Study

Author information:
Neha Christopher[1,2,] Jeanette Tamplin [3,4]

1 Graduate researcher- University of Melbourne, Melbourne, Australia.
2 Founding Board Member- Indian Association of Dance Movement Therapy, Tamil Nadu, India.
3 Senior Music Therapy Lecturer - University of Melbourne, Melbourne, Australia.
4 President - Australian Association of Music Therapy

Corresponding Author:
Neha Christopher
Email: ncchr@student.unimelb.edu.au

Statements and Declarations:
This study was funded by The Melbourne Research Scholarship for graduate researchers at The University of Melbourne.

Supplementary Document 1: This document includes data of the long answer survey responses. It begins on the next page.

Q1-

Hello! I am a PhD student at the Creative Arts Therapy Research Unit at The University of Melbourne. I am interested to gain preliminary insights on the prevalence and nature of kinesthetic empathy to foster self-regulation as a dance therapy intervention in treatment resistant depression.
This survey project has 11 questions in total.
• The first 7 questions which will ensure that you are eligible to participate in this study and will collect your contact information for correspondence. This will take around 2-3 minutes to complete.
• The next 4 questions are open ended questions and are anticipated to take 15 minutes to complete.

This project aims to collect data on:
•How kinesthetic empathy is used as a dance therapy intervention in treatment resistant depression
•Any key factor/s that support the link between the intervention and observed change, i.e., between kinesthetic empathy and self-regulation If you choose to participate in this survey project, please be informed that your participation is voluntary and will not be compensated.

Your responses will be kept completely confidential and stored securely on Qualtrics.
You have the right to withdraw at any point.

The graduate researcher (Ms. Neha Christopher) is the main point of contact and can be emailed at ncchr@student.unimelb.edu.au. The responsible researcher and primary researcher for this project is Professor Katrina McFerran.

Please note:If you feel any distress while taking this survey, please feel free to stop the survey and seek support if needed (from a friend, family member, therapist or supervisor etc.). This graduate researcher will also offer a virtual confidential conversation to debrief any content shared here by participants; details for which will be shared in a follow up email after the survey has been completed. There will be no judgment of any participants' clinical skills. These survey results will be analyzed on the foundations of respect for each participant’s work in their community.

By clicking the button below, you are acknowledging the following:
• You are at-least 18 years of age.
• Your participation in the study is voluntary.
• You are aware that you may choose to withdraw your participation at any time for any reason.
• You give consent to be contacted by the graduate researcher.
• The plain language summary and advertisement emailer provided you with adequate information to decide upon your participation in this study.

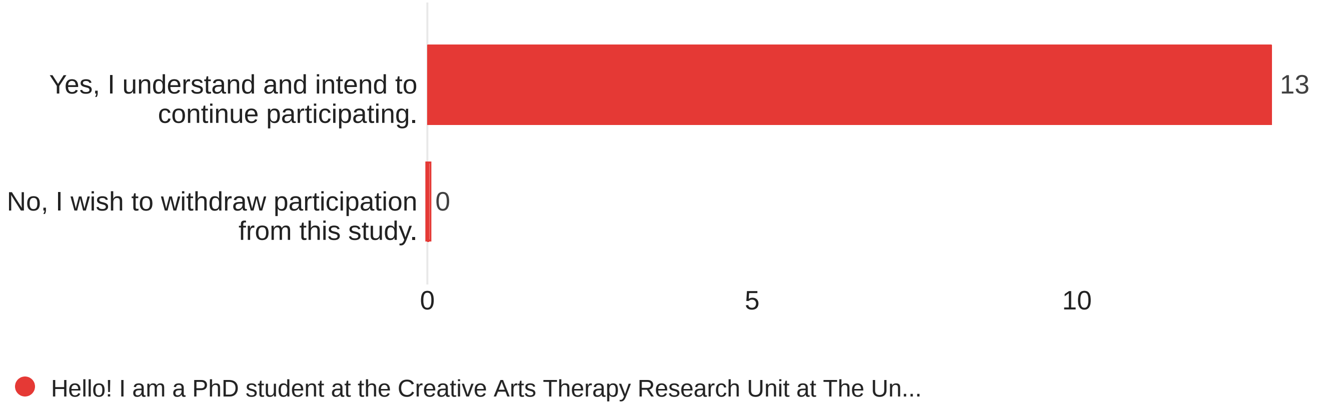

Q2-

Do you hold a Masters Degree in Dance Therapy?

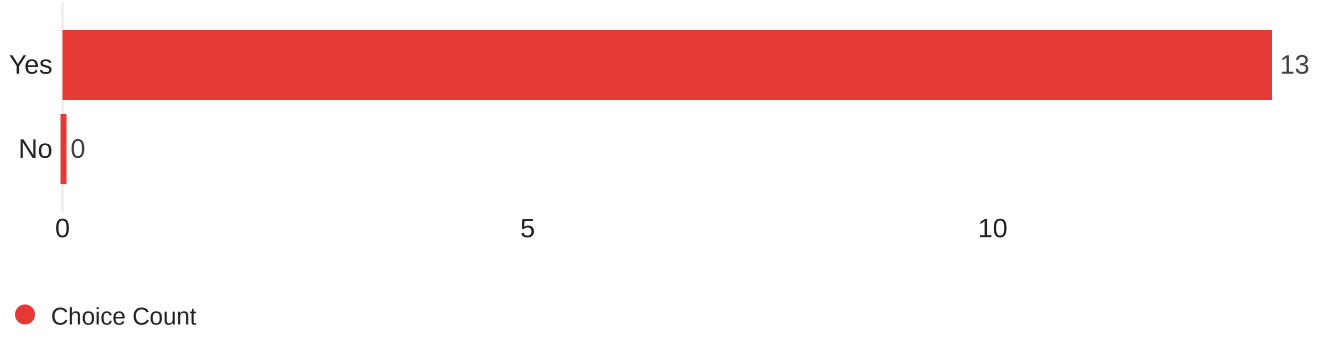

Q5-
Have you worked for a minimum of 2 years in the field of Dance Therapy? [There is no specific setting requirement; setting can vary from in-patient psychiatry setting to long term clinical settings, private practice, etc.].

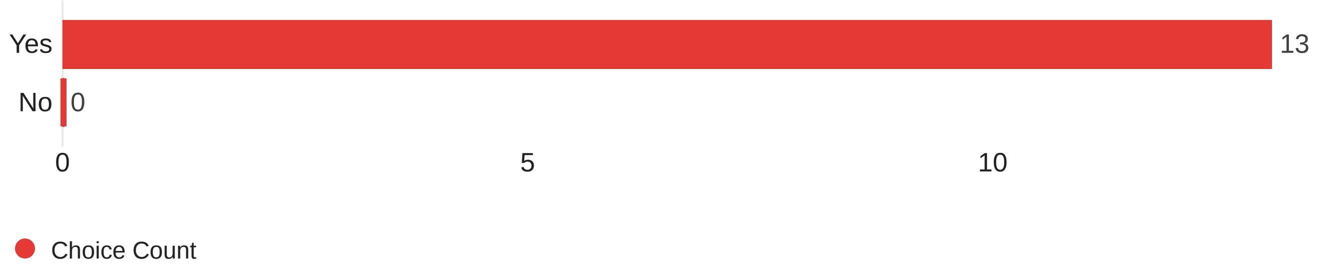

Q6-
Have you ever engaged* with clients living with treatment resistant depression?**
***In this project, engagement may be understood as an overall time period of 6 months across any time duration**
****Treatment resistant depression (TRD) refers to a type of depression where one doesn't get better in spite of intervention (Al Harbi, 2012). For example, individuals with TRD may not recover even after trying 2 cycles of anti-depressants.**

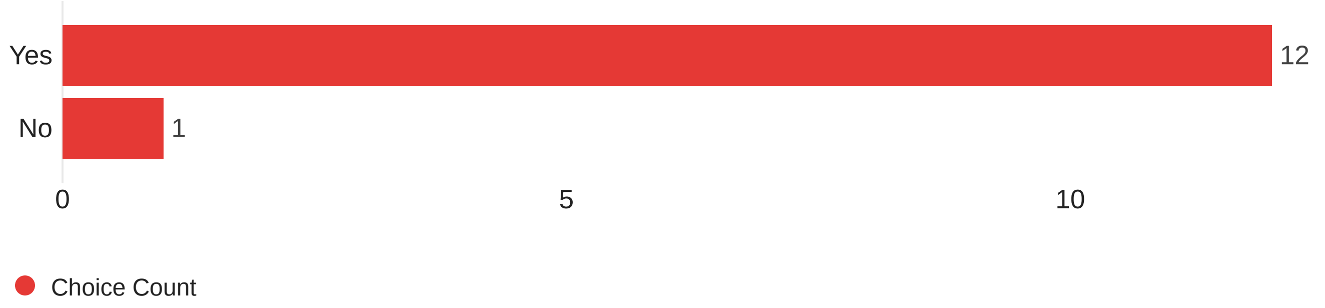

Q7-
As a dance therapist, do you/ have you employed kinesthetic empathy to foster self-regulation in your practice?

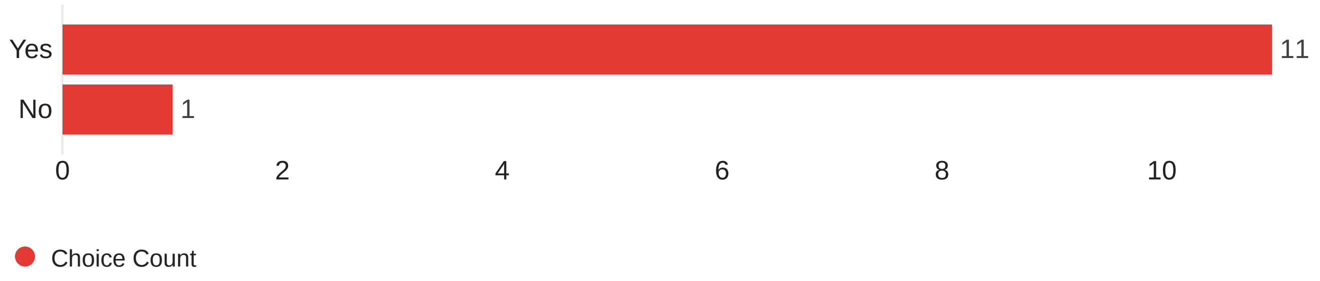

Q8-
Could you mention any specific ways in which you used kinesthetic empathy in your work?

Mirror

mirror posture, gesture, muscle tension

Through mirroring as a dmt style

kinesthetic empathy has played a huge role in fostering relational connections between the client and myself. I have also used it during the warm-up stage of the process or during the end for grounding and self-regulation purpose.

Mirroring the movement, posture and facial expression of the clients; meeting them where they're at and being curious; Being gentle and calm in nonverbal communication helping them feel safe and understood

Kinesthetic empathy is at the core of my work. I rely on the information I gather through my body, through the process of kinesthetic empathy, at all phases of my work with an individual or group. It is one of the primary ways I gather information and it is one of the primary ways I engage with my clients. Connecting via KE, helps me to attune to others, which in turn lends itself beautifully to the process of co-regulation.

Kinesthetic empathy has been a key tool to understand the embodied culture and the inner environment of my client's experience. Since many clients that do undertake dance therapy with me are coming in as a last resort as compared to all the other various psychotherapeutic interventions, it gives me an added vocabulary to understand that which they haven't been able to articulate or address in their prior attempts.

Devising any intervention required the resonant state of Kinesthetic empathy. Attuning and moving with the client in particular helped me get in this state but also over a period of time, having observed movement patters allowed this to be a continuous and consistent state with long term clients.

Q9-
In your experience, how do clients respond to kinesthetic empathy in dance therapy sessions?

Following, watching

welcoming when conscious of it, seemingly receptive when subconscious

Increased eye contact, touch or verbalization of body sensations while moving with therapist or other clients

in my experience kinesthetic empathy is used differently in individual sessions and group sessions. while in individual sessions - its more subtle and has a huge impact of small gentle movements. whereas in groups, aspects of mirroring and sharing rhythms, exploring movement and metaphor becomes more prominent. but in both scenarios, the increase in trust, communication and connection has been successful.

It takes time but this can help them start to feel comfortable; they begin to feel safe which then strengthens the therapeutic relationship; to begin to feel less resistant

In my years of experience as a DMT and working mostly with groups, I have found patients to be especially responsive to the concept of KE. I often will spend time speaking about it and teaching them to develop their own sense of KE.

In my experience, many of them are surprised with the reflections or offerings that emerge from kinesthetic empathy. They sometimes find connecting at an embodied level less effortful as compared to articulating it. The trust in the therapeutic approach of the body is deepened when they also experience it the language of the body, that which was unspoken finds its space in the relationship.

K.E. continues to be the most important factor of the relationship in DMT sessions. without it, I cannot imagine setting up a working alliance, rapport or trust.

Q10-
Please specify how you are able to attribute any mentioned changes to kinesthetic empathy.

Threw the eyes and sometimes smiling and moving tigether

I see and hear a softening, an increase in opening up verbally and emotionally-increase in emotional release.

By verbalizing what i witness within myself or from the client

There is very clear increase of trust and therefore clients become even more vulnerable and open to the connection that gets established in the sessions. This has become very obvious in these times as clients themselves talk about how distant they feel during online sessions to how connected they feel in in-person sessions. There is more willingness to bring the body to the fore during the in-person sessions.

They begin to open up more; they seem to feel like they can be more themselves; they don't seem as cautious as they did when we first began

This is a hard point to speak to, as I work in a very short term psychiatric hospital, so my time with patients is fleeting. I very much focus on the here and now and it is usually only through verbal processing and sharing, that I learn that the patient has found the process of KE to be beneficial ... or to have contributed to some shift within them.

Many a times, one can see how the body postures and movement patterns shift after experiencing Kinesthetic empathy. Clients have also mentioned how they are more comfortable with an embodied approach and open to body awareness and the language of the body as compared to the first few sessions. One of my clients used more pathways across space and their kinesphere expanded after experiencing authentic embodied empathy.

relational spaces, which are central to the therapeutic process, rely on Kinesthetic empathy to be fostered. All insight and reflection begins there.

Q11-
Based on your experience, are there any key factor/s that support the potential link between kinesthetic empathy and self-regulation?

Connection moment

I believe that when a therapist is able to relate to a client using kinesthetic empathy, the client has a felt sense of being seen/matched and creates a stronger bond/alliance between in the partnership. This then allows the client to attune to the therapist with the whole self and is moved to co-regulate.

Emotional regulation within the individual's body and mindfulness

mainly with the population that we are discussing, i have closely witnessed clients, after many months - gently start physically mirroring me eg: open posture, straighter back, eye contact, deep breathing, engaging the body in expression more and sometimes even take risks by becoming creative. though this is not an over night process, i have witnessed these changes slowly helping the client after they feel safe and settled in therapy. this has naturally led us to explore independence , and mechanism that help in self-regulation. the aspect of "here and Now" and how does one experience self-regulation has been an important part of therapy.

Co-regulation seems to be key in being able to link kinesthetic empathy and self-regulation. When we empathize with the client, we are able to co-regulate with them which can then help them self-regulate

I'm not sure I fully understand this question but I will answer as best I can. I believe that what is key, is that when I am attuning with another, through my training in KE, I am better able to offer movement interventions, designed to support or initiate self-regulation or co-regulation.

Kinesthetic empathy in my experience (reflexively and via processing) have the potential to allow for a deeper, meaningful and authentic connection with the body and how their experiences find their way through movements. This further allows the body to learn ways of feeling comfort and work towards a more tolerant embodied experience, leading to a sense of gaining resources for self- regulation

the key factor in the link would be the aspect of understanding and engaging with the client's unique body presentation and movement language to understand what self regulation means in their context
